# Research training during radiology residency: findings from the ESR Radiology Trainee Forum survey

**DOI:** 10.1186/s13244-024-01812-7

**Published:** 2024-10-07

**Authors:** Michail E. Klontzas, Martin Reim, Saif Afat, Viktoria Podzniakova, Annemiek Snoeckx, Minerva Becker, European Society of Radiology (ESR)

**Affiliations:** 1 Department of Radiology, School of Medicine, University of Crete, Heraklion, Crete Greece; 2 Department of Radiology and Interventional Radiology, Tartu University Hospital, University of Tartu, Tartu, Estonia; 3 Department of Diagnostic and Interventional Radiology, Eberhard Karls University Tuebingen, Tuebingen, Germany; 4 Department of Radiology, The Princess Alexandra Hospital NHS Trust, Harlow, UK; 5 Department of Radiology, Antwerp University Hospital and University of Antwerp, Antwerp, Belgium; 6 Faculty of Medicine and Health Sciences, University of Antwerp, Antwerp, Belgium; 7 Unit of Head and Neck and Maxilofacial Radiology, Division of Radiology, Diagnostic Department, Geneva University Hospitals, University of Geneva, Geneva, Switzerland; 8 Am Gestade 1, Vienna, Austria

**Keywords:** Survey, Research, Radiology, Training programmes, Medical education

## Abstract

**Objectives:**

To elucidate the research training exposure of radiology residents across ESR country members.

**Methods:**

A 30-question survey was constructed by the Radiology Trainee Forum and was distributed among residents and subspecialty fellows of countries members of the ESR. The survey examined the training environment, the status of research training and publications among trainees, the conditions under which research was conducted, and the exposure to activities such as grant proposal preparation and manuscript reviewing. Descriptive statistics and the chi-square test were used to assess the responses to survey questions and evaluate factors related to these responses.

**Results:**

A total of 159 participants from 29 countries provided fully completed questionnaires. Only 12/159 trainees already had a PhD degree and nearly half had never published a PubMed-indexed manuscript (76/159, 47.8%). Among those who published their papers during radiology training, most did so in the first or second year of residency (*n* = 26 and *n* = 20 participants, respectively). Most participants (79%) did not receive further statistical training during residency, fifty-five out of 159 (34.59%) respondents never had any guidance/training on how to read a paper and 58 out of 159 (36.48%) had never been encouraged to participate in any research. Most of them had worked after hours to carry out research at least a few times (47/159, 29.56%) or always (82/159, 51.57%).

**Conclusion:**

Analysis of research training among radiology trainees was performed. Areas for improvement were identified that can prompt changes in training curricula to prepare a highly competent European workforce.

**Critical relevance statement:**

This survey has identified deficits in research training of radiology residents across countries members of ESR, pinpointing areas for improvement to fortify the future of radiology in Europe.

**Key Points:**

- Research exposure and training of radiology residents varies across countries and members of ESR.
- Radiology residents largely lack systematic research training, dedicated research time, and guidance.
- Areas for improvement in research training of radiology residents have been identified, aiding the fortification of radiology research across Europe.

**Graphical Abstract:**

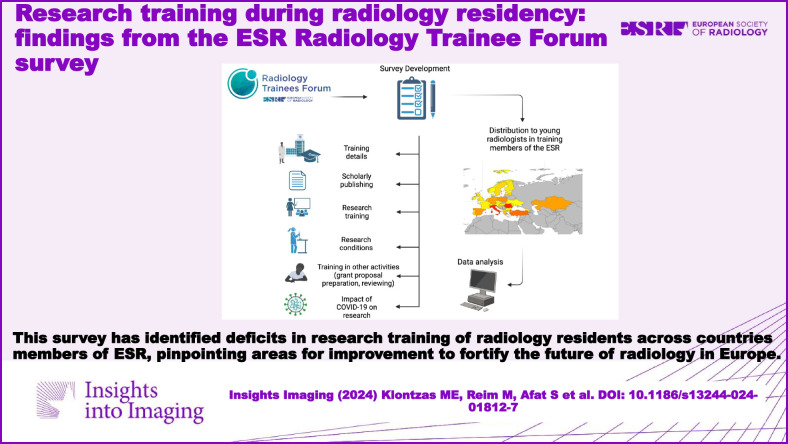

## Introduction

Radiology has entered an era of unprecedented developments, with artificial intelligence and biomedical engineering leading the advancements that impact everyday radiological practice [1, 2]. Radiologists play a crucial role in the future of the speciality, not only as users of novel technology but also as leaders of innovation, guiding developments in the field. Involvement in cutting-edge research is of utmost importance for radiologists to voice their needs and lead research teams that will produce technology relevant to everyday practice challenges.

Research exposure during speciality training varies across countries. The American Board of Radiology has established a research track residency programme for trainees with research potential, offering 18–21 months of dedicated research training during residency [3]. Similarly, the National Institute of Health and Care Research in the United Kingdom, funds Clinical Lecturer positions for radiology trainees, securing teaching responsibilities and dedicating 50% of research time for smaller research projects or higher degree pursuits (e.g., a doctorate) [4].

Currently, section B-I-18 of the Level I European Training Curriculum for radiology, developed by the European Society of Radiology (ESR) is dedicated to research training and Level II of the same curriculum explicitly states that “For interested trainees, options to actively perform radiological research projects should ideally be offered” [5]. However, significant variability exists in local curricula allowing different levels of research exposure for radiology residents. In addition, the European School of Radiology (ESOR) has created a dedicated course entitled “Fundamentals of Radiological Research”, available through the ESR premium education package [6]. Nonetheless, the involvement of radiology residents in radiological research varies among hospitals and countries, and the current status of research exposure and understanding has not been recently assessed.

To elucidate the research training exposure of radiology residents across ESR country members, the Radiology Trainee Forum (RTF) under the auspices of the education committee of ESR, created a dedicated survey. The survey was administered across ESR member countries to investigate the current status of research training among radiology residents and to ultimately identify areas for future improvement.

## Materials and methods

### Participants and survey distribution

**Fig. 1:**
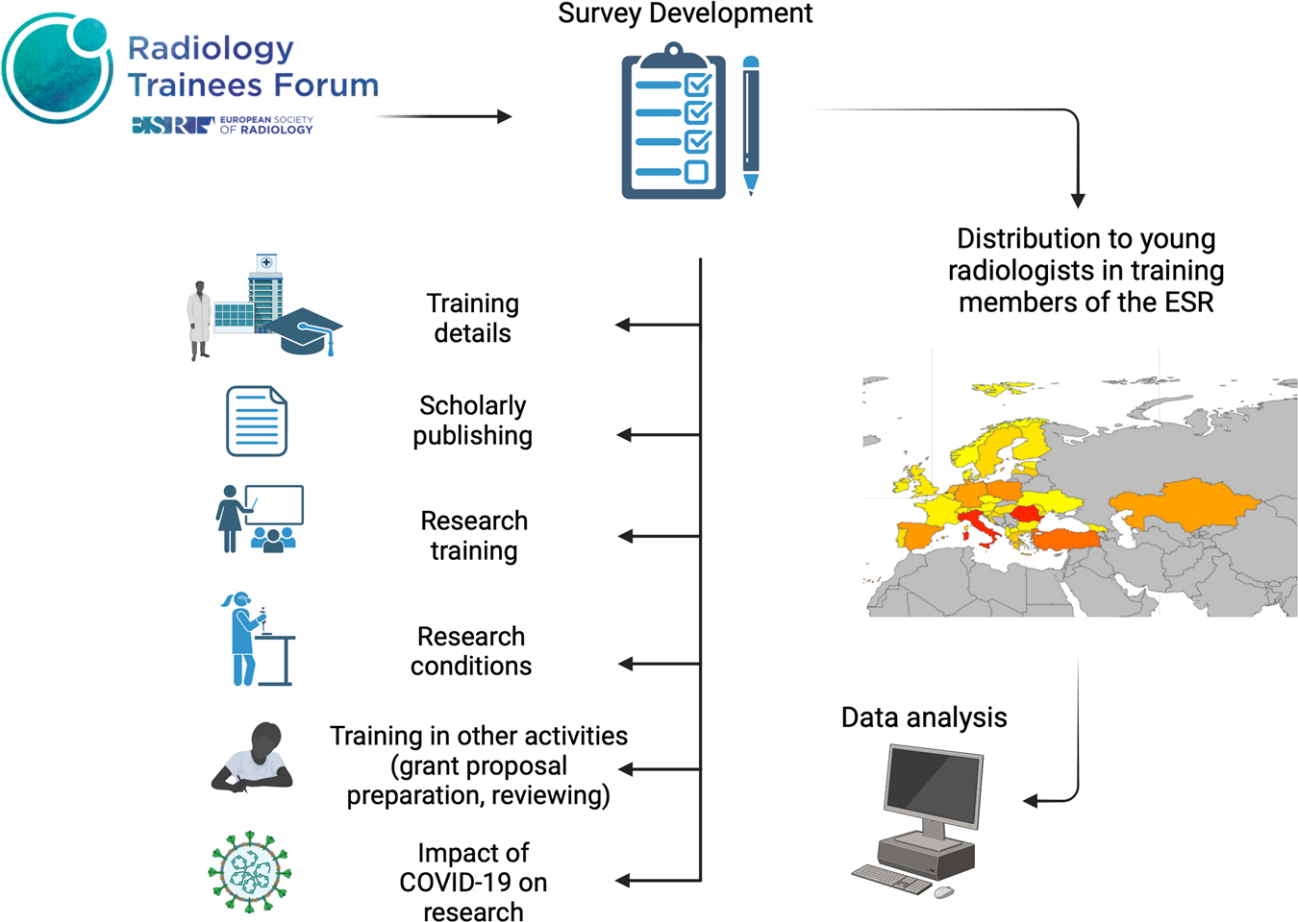
Flowchart demonstrating the process of survey construction, administration and analysis

A survey consisting of 30 questions was developed by the RTF board members to assess the current status of research training among radiology residents in member countries of ESR. The survey was distributed by ESR to all registered members in training including subspecialty fellows, using SurveyMonkey. It was open for two weeks and responses were automatically stored upon completion. Ethics approval was not required for this survey. The methodology followed in this study is illustrated in Fig. 1.

### Survey structure

The initial questions of the survey addressed the country and year of training, and the type of hospital where training was conducted (University Hospital or General Hospital). Participants were also asked whether they possess a Doctor of Philosophy (PhD) and whether this was obtained before, during, or after residency. Subsequent questions focused on the number of publications, the year of training when their first PubMed-indexed paper was published, and whether this was a requirement to complete their radiology training. The survey then explored the training participants received in areas such as statistics, research methodology, manuscript preparation, grant proposal preparation, scholarly reviewing and the encouragement by senior colleagues to publish. It also inquired about participant’s willingness to undertake formal training in research methodology. To evaluate the workload related to research activities, participants were asked about the presence of dedicated research time during training, the availability of salary funding for research activities and the impact of the COVID-19 pandemic on their research output. A list of the questions can be found in Supplementary File 1.

### Statistical analysis

Descriptive statistics were used and responses to closed-type questions were recorded in a categorical scale and were expressed as frequencies or percentages. The relationship between the number of publications and other factors was compared with the use of the chi-square test. *p*-values less than *a* = 0.05 were considered significant. Analyses were performed with the use of SPSS v29 (IBM, Armonk NY, USA).

## Results

**Fig. 2:**
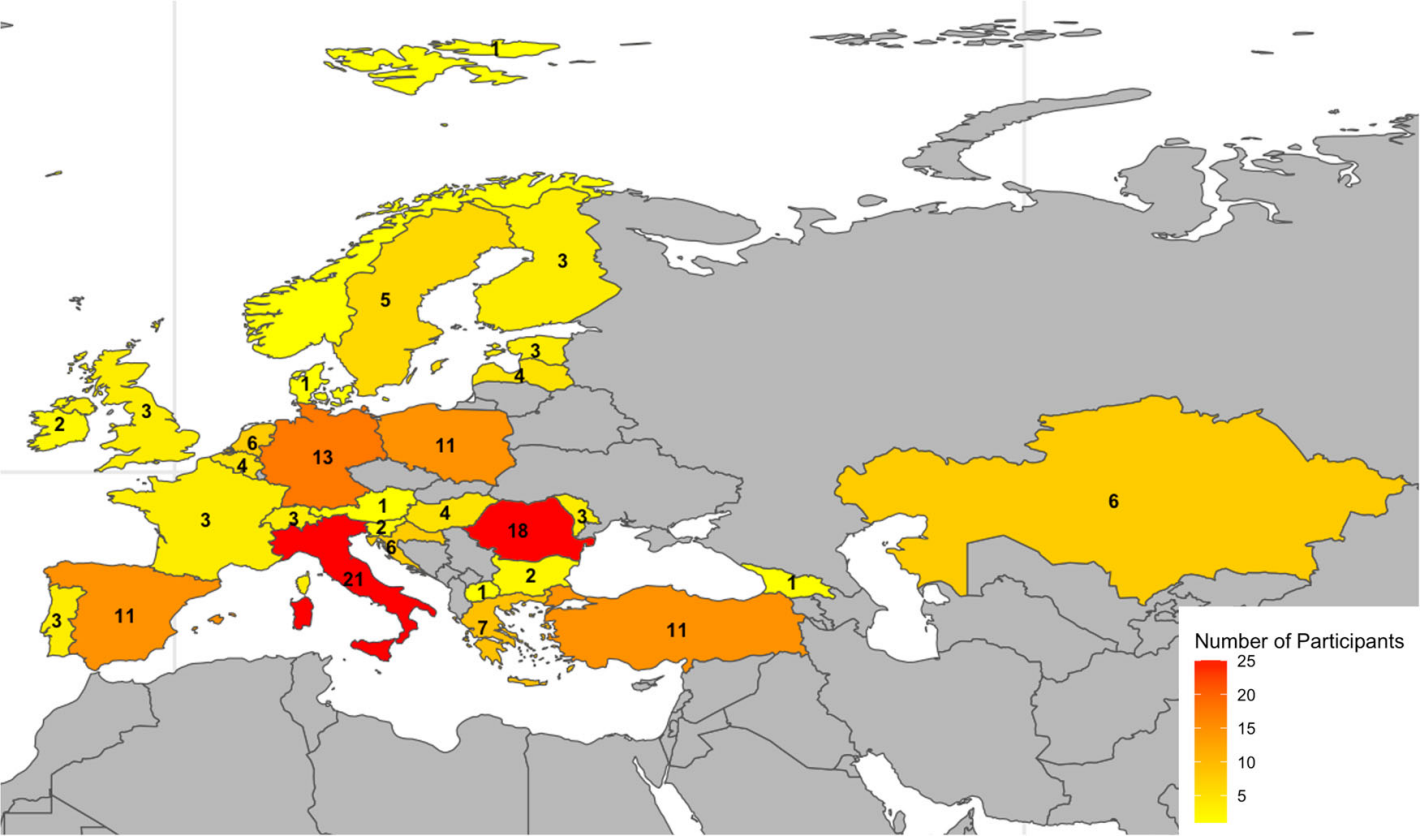
Map demonstrating the distribution of survey respondents across countries members of the ESR

A total of 159 participants from 29 countries provided fully completed questionnaires. Italy contributed the majority of responses (*n* = 21), followed by Romania (*n* = 18) and Turkey (*n* = 11). The geographical distribution of responses is shown in Fig. 2. Out of 159 participants, a total of 22 (13.8%), 35 (22%), 38 (23.9%), 37 (23.3%), 20 (12.6%) and 7 (4.4%) were first, second, third, fourth, fifth-year trainees and fellows, respectively. The majority of respondents worked at university hospitals (*n* = 128, 80.5%) compared to general hospitals (*n* = 31, 19.5%).

Only 12/159 (7.5%) trainees had already been awarded a PhD degree, with seven obtaining the PhD before, two during and three after residency. Nearly half of the respondents had never published a PubMed-indexed manuscript (76/159, 47.8%), 62 (39%) had published 1–5 manuscripts, 12 (7.5%) had published 6–10 manuscripts, 4 (2.5%) had published 11–15 manuscripts, and 5 (3.1%) had published more than 15 PubMed-indexed manuscripts. The number of published manuscripts was significantly related to the possession of a PhD degree (*p* < 0.001) but did not show a statistically significant relationship with the type of hospital (*p* = 0.317, University vs General Hospital). The majority of participants had participated in clinical research 86/159 (54.1%), compared to 8/159 (5%) who participated in experimental, whereas 20/159 (12.6%) had participated in both research types.

Twenty-nine of the respondents (29.6%) indicated that publication of manuscripts is compulsory during residency. Importantly, the number of published papers was not significantly related to the requirement to publish (*p* = 0.775). Among those who published their papers during radiology training, most did so in the first or second year of residency (*n* = 26, 16.35% and *n* = 20, 12.58% participants, respectively), with the number decreasing in subsequent years (*n* = 7, 7, 1 and 2 for the third, fourth, fifth year, and subspecialty fellows, respectively).

**Fig. 3:**
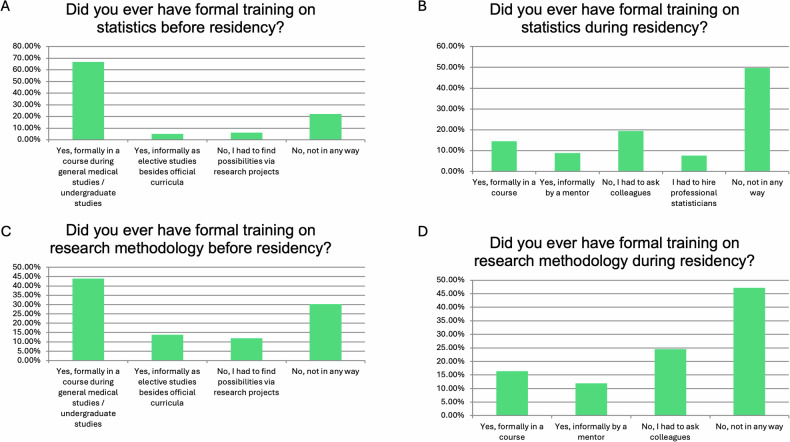
Responses to questions related to statistical and research training. Graphs **A**–**D** present the responses to individual questions

Regarding training in statistics and research methodology, the majority 106 (66.67%) had received statistical training before residency during medical school or undergraduate studies. However, half of the participants 79 (49.69%) did not receive further statistical training during residency (Fig. 3A, B). Similar responses were obtained when participants were asked about training in research methodology (Fig. 3B, C).

**Fig. 4:**
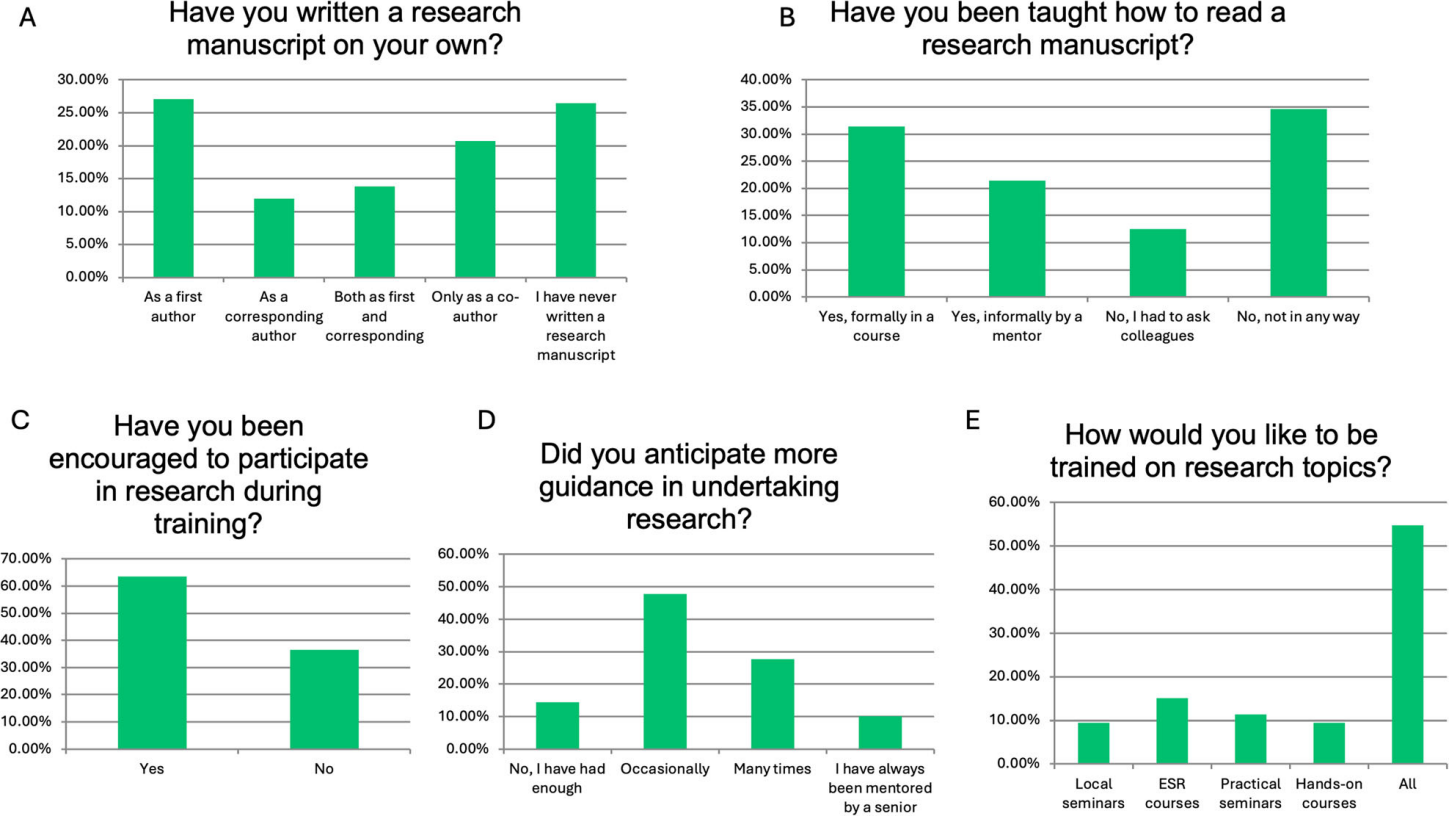
Responses to questions related to training on manuscript preparation and guidance during research. Graphs **A**–**E** present the responses to individual questions

Participants were also asked about their experience and guidance in writing manuscripts. Among respondents, 43 (27.04%) had published a manuscript as first authors followed by 33 (20.75%) participants who had a publication only as co-authors. Notably, 55 out of 159 (34.59%) respondents never had any guidance/training on how to read a paper and 58 out of 159 (36.48%) had never been encouraged to participate in any research. The majority expected at least occasional guidance (*n* = 76, 47.8%) and expressed a desire to be trained through various means, including local seminars, ESR courses, practical seminars, and hands-on courses (Fig. 4).

**Fig. 5:**
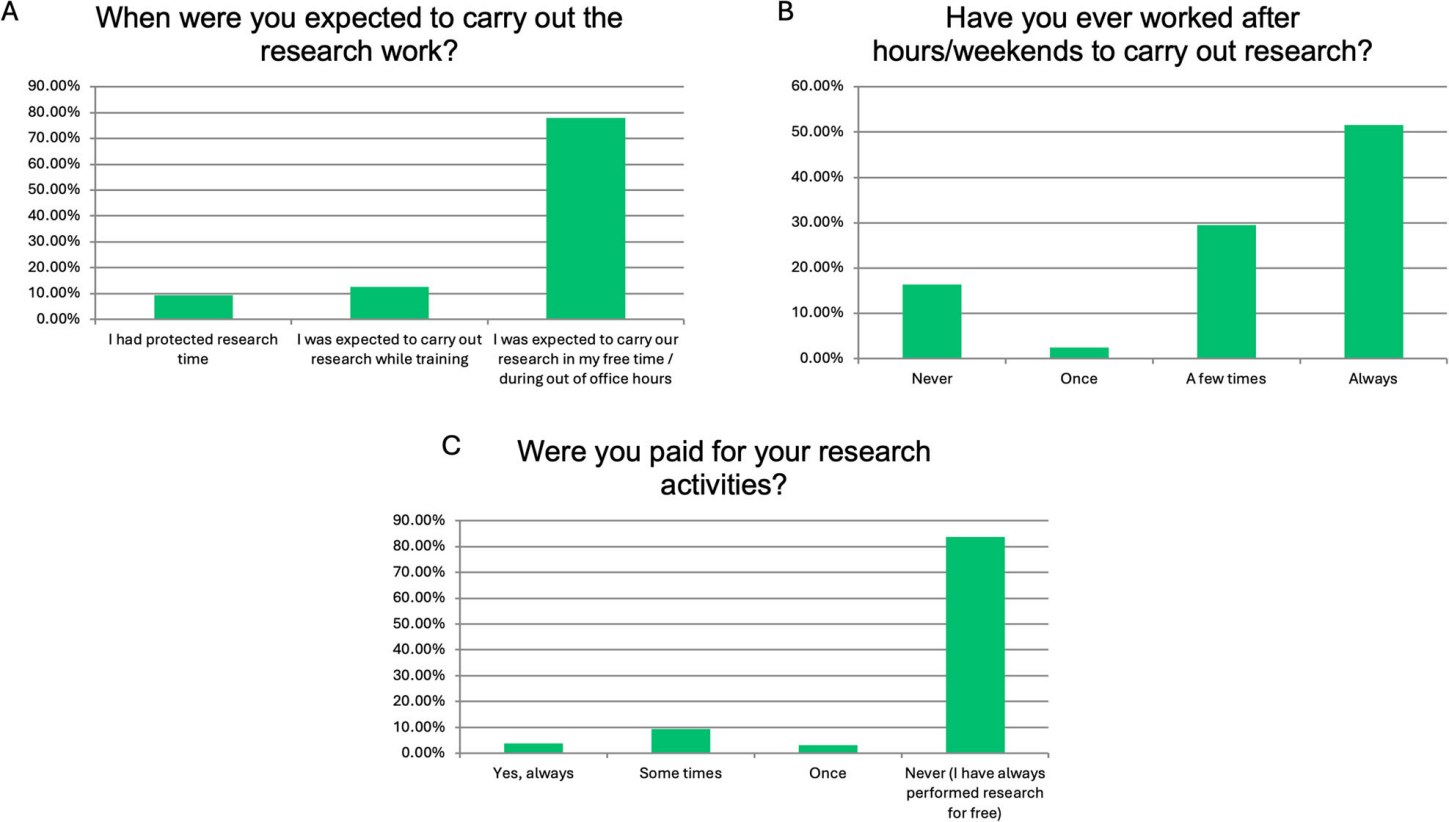
Responses to questions related to conditions when undertaking research. Graphs **A**–**C** present the responses to individual questions

**Fig. 6:**
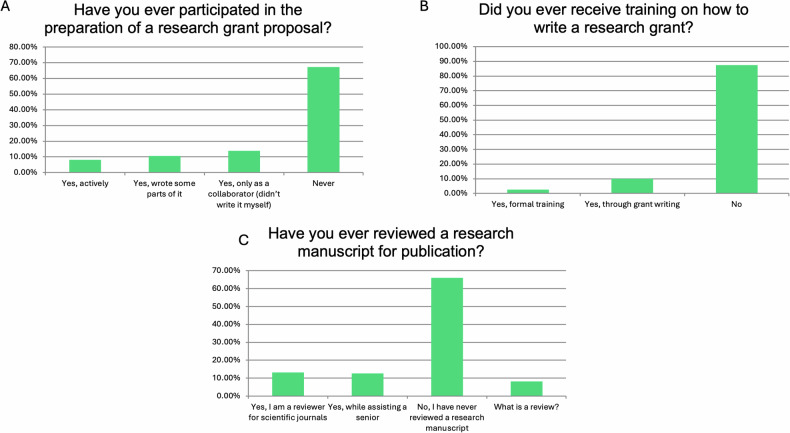
Responses to questions related to grant proposal preparation and manuscript review. Graphs **A**–**C** present the responses to individual questions

Regarding the time spent in research, most trainees (124/159, 77.99%) were expected to undertake research in their free time. Most of them had worked after hours to carry out research at least a few times (47/159, 29.56%) or always (82/159, 51.57%). The majority of this research (133/159, 83.65%) was performed without any payment for these activities (Fig. 5). In addition, most of them had never participated in grant proposal preparation (107/159, 67.3%) and had never received any training to do so (139/159, 87.42%) (Fig. 6).

When asked about the effect of the COVID-19 pandemic on their research activities, half of the participants replied that it did not affect them (79/159, 49.69%), some claimed they were negatively affected (54/159, 33.96%) and a few reported a positive effect (26/159, 16.35%). For the majority of trainees, the pandemic did not affect their research plans (109/159, 73.15%) or their research training (115/159, 77.70%) (Supplementary Fig. 1).

## Discussion

In this study, we conducted an ESR-wide survey to evaluate the status of research training and involvement in research activities among radiology residents and fellows. The survey results revealed important facts on the research experience of radiology trainees, indicating that half of the respondents had never published a research manuscript, and only a minority had published a first-author manuscript. Importantly, research activities were often conducted in their free time without dedicated research time during training, and in most cases, were unpaid. Additionally, trainees generally lacked experience in grant proposal preparation and manuscript review.

Half of the respondents had never published a manuscript, irrespective of the type of hospital that they were trained in (University vs General Hospital). This highlights a significant deficiency in exposure to academic work. As pointed out by Jenkins et al [7], research is the “bedrock of evidence-based practice”, and is closely linked to leadership skills and career development. This is important when comparing the radiology workforce to that in the US, where diagnostic radiology trainees typically had an average of 8 abstracts/publications, and interventional radiology trainees had 12 abstracts/publications before starting their residency [8]. Our results indicate that publication numbers were higher among trainees who had completed a PhD degree, although fewer than 10% of trainees held such a degree.

Our findings indicate that a very low number of trainees have a PhD degree. This is important since the possession of a PhD degree is, in most cases, a requirement for academic positions and allows for the nurturing of independent researchers. Nonetheless, it needs to be considered that in certain countries, residents may have limitations in applying for a PhD. For example, in Italy, up to 2022, only last year residents could start a PhD (which typically lasts for 3 years). A new law in 2022 removed this limitation (but the first residents could only complete a PhD in 2025, so not in time for the survey we have conducted). In these cases, the PhD can either be performed before or after the residency. This needs to be taken into account since Italy was one of the countries with the most respondents in our survey. Important discrepancies in training also exist in terms of fellowships which do not officially exist in countries such as Greece or Italy, whereas they are to a certain extent incorporated within radiology training in other countries such as the United Kingdom. These discrepancies in training will inevitably lead to discrepancies in research exposure. This calls for the standardisation of radiology training across ESR member countries, allowing for the horizontal introduction of official research components within residency training that will improve the research landscape within Europe.

Making research attractive to trainees is directly related to the conditions under which it is offered. Our results show that research is usually expected to be carried out in their free time and without any financial compensation. This issue has been addressed in a limited number of countries such as the UK, where academic residency posts have been created to allow for dedicated paid research time during residency, particularly for those pursuing higher degrees [4]. However, this is not the case in the majority of national training programmes. This situation appears unchanged over the past decades, as indicated by a survey conducted more than 30 years ago among radiology department chairs. This survey showed that only half of the examined training programmes offered some formal research training and that only a minority of trainees were interested in additional research activities [9]. Providing dedicated research time could incorporate courses on research methodology, potentially increasing the low rates of training in manuscript preparation, grant proposal preparation, scholarly reviewing, and research methodology as found in our results. ESOR courses on research methodology could form the basis of this training, but residents are unlikely to participate in such courses in cases where research is carried out for free and the cost for such courses is not reimbursed. A summary of areas for improvement and potential solutions has been provided in Supplementary Table 1.

Our results also showed that the COVID-19 pandemic negatively impacted the research activities of one-third of the participants. As shown in previous research of the RTF and the ESR education committee [10], most residents were not allowed to change from clinical work to research during the pandemic, which can explain the retrospectively identified negative impact of the pandemic on clinical work.

## Conclusion

Research training during radiology residency is crucial for fostering innovation in radiology and shaping future leaders in the field. The survey results identified areas for improvement that can prompt changes in training curricula to prepare a highly competent European workforce. These areas include training on research methods, support for research during training, training on grant proposal writing, and manuscript reviewing. Ultimately, equipping radiologists in training with advanced research skills will build a community capable of advancing the field and securing the future of our speciality.

## Supplementary information

ELECTRONIC SUPPLEMENTARY MATERIAL

## Data Availability

All data necessary to replicate the results of this survey have been reported in this manuscript.

## Abbreviations

ESOR: European School of Radiology
ESR: European Society of Radiology
PhD: Doctor of Philosophy
RTF: Radiology Trainee Forum
