# Supplementary material for: Research training during radiology residency: findings from the ESR Radiology Trainee Forum survey
Source: Insights Imaging. 2024 Oct 7;15:236. doi: 10.1186/s13244-024-01812-7 (PMC11458849; doi:10.1186/s13244-024-01812-7)
Supplement: Supplementary file 1 — ELECTRONIC SUPPLEMENTARY MATERIAL [file 13244_2024_1812_MOESM1_ESM.pdf]

Research training during radiology residency: findings from the ESR Radiology Trainee Forum survey
ELECTRONIC SUPPLEMENTARY MATERIAL

	Question
1	Where are you from?
2	What year of radiology training are you in?
3	Do you work at a University Hospital?
4	Do you already have a PhD?
5	When did you obtain your PhD?
6	How many PubMed-indexed manuscripts have you published?
7	At which year of residency did you publish your first radiology manuscript?
8	Are you required to publish a manuscript during the period of your residency?
9	What type of radiology research have you worked on?
10	Did you ever have formal training on statistics during residency?
11	Did you ever have formal training on statistics before residency?
12	Did you ever have formal training on research methodology during residency?
13	Did you ever have formal training on research methodology before residency?
14	Have you been taught how to read a research manuscript?
15	Have you written a research manuscript on your own?
16	Have you been encouraged to participate in research during training?
17	When were you expected to carry out the research work?
18	Have you ever worked after hours/weekends to carry out research?
19	Were you paid for your research activities?
20	Did you anticipate more guidance in undertaking research?
21	Have you ever participated in the preparation of a research grant proposal?
22	Did you ever receive training on how to write a research grant?
23	Have you ever reviewed a research manuscript for publication?
24	How would you like to be trained on research topics?
25	Has the pandemic (COVID-19) affected research in your institution?
26	Please explain how:
27	Has the pandemic (COVID-19) affected your research plans?
28	Please explain how:
29	Has the pandemic (COVID-19) affected your research training?
30	Please explain how:

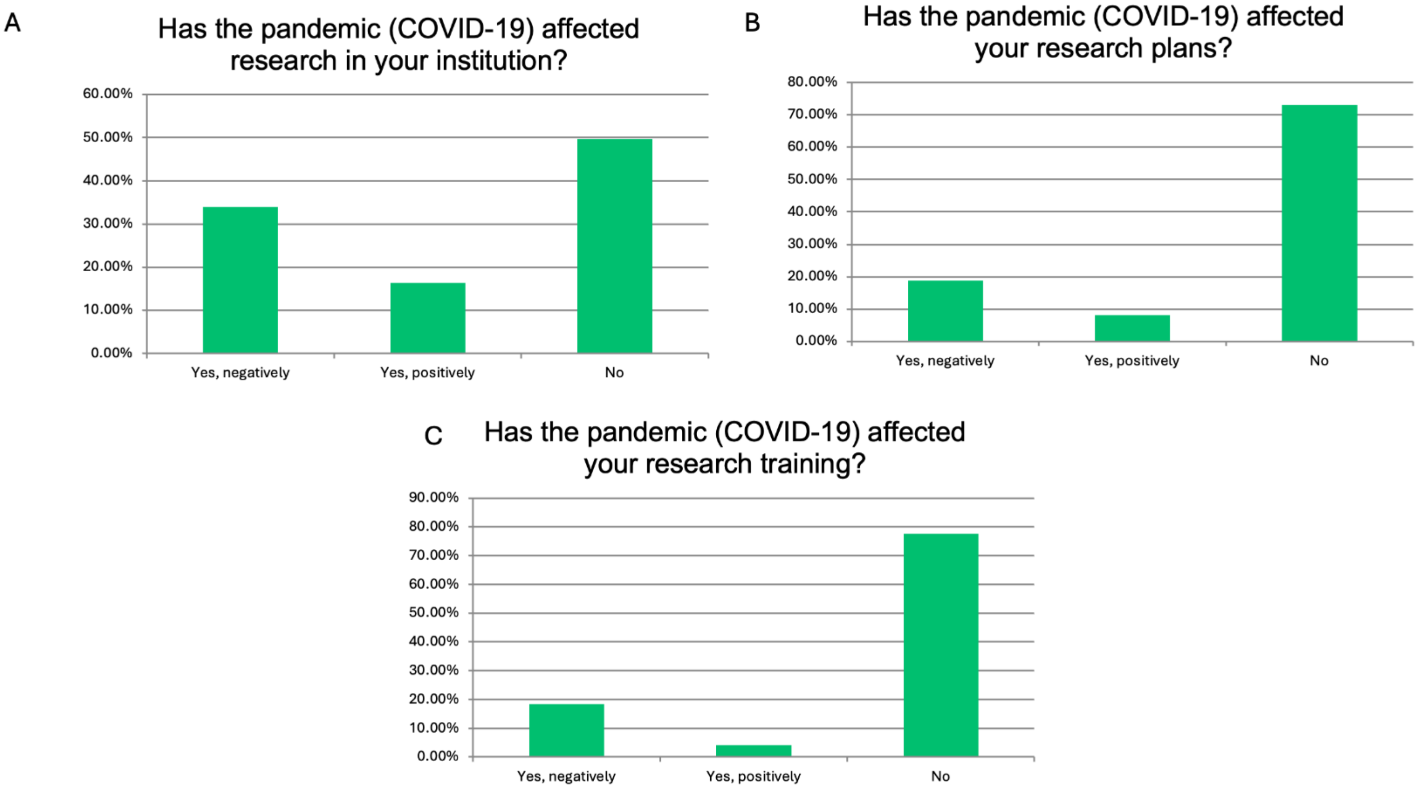

Supplementary Fig. 1 Responses to questions related to the effect of COVID-19 pandemic on research activities of radiology residents

Supplementary Table 1. Areas for improvement and potential measures

Areas for improvement	Potential measures
**Research During Training**	Allocate specific periods within the training schedule dedicated to research activities.
	Offer funding opportunities or grants specifically aimed at supporting trainee-led research.
	Offer statistical modules to trainees during the residency.
**Grant Proposal Training**	Create comprehensive training modules focused on the various aspects of grant proposal preparation.
	Organize workshops and seminars led by experienced researchers and grant writers.
	Integrate grant writing into the residency program to foster a research-oriented culture.
**Support and Mentorship**	Offer guidance and support with experienced mentors who can provide advice on project design, data analysis, and manuscript preparation.
	Provide access to research databases, software, and other essential tools.
	Create support networks and forums where trainees can share experiences, seek advice, and collaborate on projects.
